# Combining Behavioral Economics–Based Incentives With the Anchoring Strategy: Protocol for a Randomized Controlled Trial

**DOI:** 10.2196/39930

**Published:** 2023-04-28

**Authors:** Chad Stecher, Sara Cloonan, Sebastian Linnemayr, Jennifer Huberty

**Affiliations:** 1 Arizona State University Phoenix, AZ United States; 2 RAND Corporation Santa Monica, CA United States; 3 Fit Minded LLC Phoenix, AZ United States

**Keywords:** habits, mindfulness meditation, mobile apps, mobile health, mHealth, adherence, meditation, mindfulness, intervention, apps, smartphone apps, stress, management, mobile phone

## Abstract

**Background:**

Chronic (ie, long-term) elevated stress is associated with a number of mental and physical health conditions. Mindfulness meditation mobile apps are a promising tool for stress self-management that can overcome several barriers associated with in-person interventions; however, to date, poor app-based intervention adherence has limited the efficacy of these mobile health tools. Anchoring, or pairing, a new behavior with an existing routine has been shown to effectively establish habits that are maintained over time, but this strategy typically only works for those with high initial motivation and has yet to be tested for maintaining meditation with a mobile app.

**Objective:**

This study will test novel combinations of behavioral economics–based incentives with the anchoring strategy for establishing and maintaining adherence to an effective dose of meditation with a mobile app.

**Methods:**

This 16-week study will use a 5-arm, parallel, partially blinded (participants only), randomized controlled design. We will implement a fractional factorial study design that varies the use of self-monitoring messages and financial incentives to support participants’ use of their personalized anchoring strategy for maintaining adherence to a ≥10 minute-per-day meditation prescription during an 8-week intervention period, followed by an 8-week postintervention observation period. Specifically, we will vary the use of self-monitoring messages of either the target behavior (ie, meditation tracking) or the outcome associated with the target behavior (ie, mood symptom tracking). We will also vary the use of financial incentives conditional on either meditation at any time of day or meditation performed at approximately the same time of day as participants’ personalized anchors.

**Results:**

Continuous meditation app use data will be used to measure weekly meditation adherence over the 16-week study period as a binary variable equal to 1 if participants complete ≥10 minutes of meditation for ≥4 days per week and 0 otherwise. We will measure weekly anchoring plan adherence as a binary variable equal to 1 if participants complete ≥10 minutes of meditation within +1 or −1 hour of the timing of their chosen anchor on ≥4 days per week and 0 otherwise. In addition to these primary measures of meditation and anchoring plan adherence, we will also assess the secondary measures of stress, anxiety, posttraumatic stress disorder, sleep disturbance, and meditation app habit strength at baseline, week 8, and week 16.

**Conclusions:**

This study will fill an important gap in the mobile health literature by testing novel intervention approaches for establishing and maintaining adherence to app-based mindfulness meditation. If successful, this study will identify an accessible and scalable stress self-management intervention that can help combat stress in the United States.

**Trial Registration:**

ClinicalTrials.gov NCT05217602; https://clinicaltrials.gov/ct2/show/NCT05217602

**International Registered Report Identifier (IRRID):**

DERR1-10.2196/39930

## Introduction

### Problem Statement

The prevalence of stress in the United States is at an all-time high, with >75% of US adults reporting elevated levels of stress in 2021 [1,2]. Chronic (ie, long-term) elevated stress is associated with a number of mental and physical health conditions, such as depression, anxiety, cardiovascular disease, and reduced cognitive functioning [3-5]. The COVID-19 pandemic exacerbated stress levels, with 78% of Americans reporting that the pandemic was a significant stressor in their lives and 67% of Americans experiencing significantly increased stress over the course of the pandemic [6]. Recent reports by the American Psychological Association have documented the cumulative negative effects of increased stress during the pandemic on mental and physical health, where 84% of Americans in 2021 reported feeling at least 1 emotion associated with prolonged stress, such as anxiety, sadness, or anger [2,7]. In addition, the reported physical health consequences of chronic elevated stress during the pandemic include undesired weight changes, sleep problems, and increased alcohol consumption [2,7].

These findings demonstrate the significant consequences that chronic elevated stress can have on health outcomes, and existing research has shown that these negative health effects also impose a large economic burden. Recent reports suggest that stress is associated with annual costs of >US $300 billion owing to workplace absenteeism, accidents, reduced productivity, and employee turnover, in addition to direct medical, legal, and insurance costs [8]. Despite the clear need for stress management resources, only 54% of Americans reported seeking help for their mental health in 2020 [9], and nearly half of Americans delayed or canceled health care service appointments after the beginning of the pandemic [7]. Thus, there is an urgent need for novel stress self-management solutions that are more accessible and that can provide alternative approaches for stress reduction than those currently offered by conventional mental health care in the United States.

The rapid development and widespread availability of mobile health (mHealth) apps for treating mental health conditions may be a key self-care solution to address the ongoing stress epidemic. Mindfulness meditation apps are a particularly promising tool for stress self-management, as they can overcome several barriers associated with in-person interventions, such as cost, stigma, and accessibility [10]. Importantly, previous research has demonstrated the efficacy of app-based mindfulness meditation interventions for improving mental health outcomes, such as depression, anxiety, and sadness, compared with both control groups and traditional in-person interventions [10]. In addition, our own work with the Calm meditation app, one of the most popular commercially available mindfulness meditation apps [11], has shown that meditation with Calm can improve a wide range of mental health outcomes and reduce stress [12-14].

Despite these promising findings, many mHealth app–based studies have reported significant dropout rates and poor intervention adherence (intervention adherence rates ranging from 39% to 92%), which ultimately limit the efficacy of app-based meditation interventions for stress management [15,16]. In general, user retention rates for mHealth apps are low, with one systematic review finding median 15- and 30-day mental health app retention rates of 4.9% and 4.7%, respectively [17]. This pattern of declining user engagement with apps has been frequently observed in the mHealth literature [18-20] and mirrors the decline in treatment adherence documented in other behavioral interventions and clinical settings [21-23]. Although a growing field of research has started to examine the issue of maintaining adherence to app-based interventions and other behavioral and medical treatments [19,24], little is known about the strategies that can successfully maintain adherence in these settings. Thus, there exists a critical need for researchers to identify and implement novel, effective, and accessible strategies for maintaining adherence to health apps, particularly meditation apps, that can help combat the alarming rates of elevated stress in the United States.

### Study Aims

This study will test novel combinations of behavioral economics–based incentives with a habit formation intervention from the psychology literature for establishing and maintaining adherence to an effective dose of meditation with a mobile app. In a 5-arm randomized controlled trial, we will implement a fractional factorial study design that varies the use of self-monitoring messages and financial rewards to support participants’ use of the anchoring strategy for maintaining adherence to a ≥10 minute-per-day meditation prescription during an 8-week intervention period, followed by an 8-week postintervention observation period. The study is designed to examine 3 specific study aims. First, we will assess the study group differences in adherence to daily meditation prescriptions during the 8-week intervention. Second, we will estimate the study group differences in meditation adherence maintenance over the 8-week postintervention observation period. Third, we will investigate the role of anchoring performance during the intervention on participants’ ability to maintain their meditation app adherence during the postintervention observation period. In this paper, we will outline previous research on mobile meditation apps, the background and rationale behind the adherence strategies we developed, our research methodology, and our hypotheses.

### App-Based Mindfulness Meditation: Strengths and Weaknesses

Previous research has demonstrated the effectiveness of mindfulness meditation interventions in improving a wide range of health outcomes in both clinical and nonclinical populations, including stress, anxiety and depressive symptoms, well-being, and sleep [15,16,25]. While traditional mindfulness meditation interventions have been delivered via in-person sessions, app-based interventions have become increasingly popular in recent years because they are less costly and more accessible than face-to-face interventions [15,16]. In addition, a growing body of research has established the feasibility and acceptability of app-based mindfulness meditation interventions [26,27], suggesting that they are scalable and acceptable alternatives to traditional in-person interventions [12,13,16,28]. Several randomized controlled trials and meta-analyses have also documented the efficacy of app-based interventions on various mental health outcomes, including stress reduction, finding similar effects when compared with in-person interventions and when compared with control groups [12,16,29]. Many of these effects have also been observed during the COVID-19 pandemic [29], suggesting that meditation apps may be a useful self-management tool for reducing stress during a prolonged period of stress, such as the pandemic.

Despite these promising findings, the efficacy and generalizability of app-based interventions are limited by their substantial attrition and poor adherence. Previous meta-analyses have estimated app-based intervention study attrition rates to range from 31% to 42% and intervention adherence rates (ie, the number of sessions actually completed compared with the prescribed amount) to range from 39% to 92% [15,16,30]. Baumel et al [17] examined objective user adherence with various mental health Android apps, including those specifically for mindfulness meditation, and found that only a small portion of users actually used the apps for an extended period, with open rates declining >80% between days 1 and 30 across all mental health apps. For mindfulness meditation apps specifically, median 15- and 30-day retention rates were 4.9% and 4.7%, respectively [17], greatly limiting the efficacy of these mHealth tools. Similar patterns of declining adherence have been observed in our research with the Calm meditation app, in which adherence rates to the daily prescription of ≥10 minutes of meditation with Calm fell by approximately 68% over the course of a 16-week study period [31]. This pattern of rapidly declining meditation app use limits the impact of these mental health solutions. The next step in the mHealth research is therefore to find effective interventions to keep mobile app users engaged, that is, to maintain meditation app adherence.

In general, study attrition and adherence are critical issues in mHealth interventions that must be addressed to improve the efficacy of these therapeutic tools. Health behavior models such as the transtheoretical model [32,33] outline several key stages of behavior adoption that should be considered when developing interventions: precontemplation, contemplation, preparation, action, and maintenance stages. Individuals who download a health app and purchase a subscription are likely to be in the preparation or action stage because they have taken steps to initiate behavior change. However, common app-based mHealth issues, such as poor adherence, suggest that significant barriers exist when moving from the preparation and action stages to the maintenance stage. The proposed socio-cognitive intervention strategies have focused on bolstering self-efficacy for dealing with obstacles, increasing motivation, and reiterating the long-term benefits of performing a behavior; however, to date, these strategies have largely been unsuccessful in maintaining health behavior changes [34-38]. Thus, in this study, we synthesized theory- and evidence-based intervention approaches from psychology and behavioral economics to test novel combined strategies for maintaining adherence to meditation using the accessible and scalable Calm app.

### Habits as a Key Strategy to Maintain Adherence

Psychological research suggests that developing a habit may be a successful behavioral strategy for maintaining behaviors over time [39]. Habits have been shown to underlie many long-term healthy behaviors [40] and may be a key strategy for maintaining adherence to a mindfulness meditation app [31,41]. Existing research has shown that habits are formed gradually over time as an individual repeatedly and consistently performs a behavior in response to the same stimulus or contextual cue, to the point where it can be performed automatically with little or no cognitive effort [23,41,42]. Through such “context-dependent repetition,” [43] exposure to the cue reflexively, or nonconsciously, triggers the initiation of the behavior [44]. In this way, behavioral control is delegated to external cues, thereby reducing the demand on an individual’s attention and memory processes [42,45,46]. Importantly, habits have been shown to persist long term despite declining motivation or willpower [47-49] and even when participants were distracted [50]. In addition, habit formation strategies have been used to successfully maintain various health behaviors, such as dietary habits [51,52], physical activity [53-56], medication adherence [57-60], and dental hygiene [61,62], among others [63,64].

One common intervention strategy for promoting habit formation is anchoring, which is when a new behavior is paired, or “anchored,” to an existing routine (ie, the contextual cue) [23]. Anchoring has successfully established health habits, such as increased physical activity [65], improved diet [51,66-68], smoking cessation [69], and medication adherence [70,71]. However, it takes approximately 3 months of adherence to an anchoring plan to form a habit [43,72-74], and many participants fail to adhere to their anchoring plan for this entire habit formation period [23,75,76]. Common barriers to anchoring plan adherence include lack of motivation [77,78] and self-efficacy [79,80] and forgetfulness [81]; on average, only 55% of anchoring intervention participants stick with anchoring long enough to form a habit. In a recent study, using the anchoring plan alone did not lead to meditating for more days per week over a 3-month intervention [82], which further suggests that additional strategies may be needed to increase anchoring plan adherence for establishing a new meditation habit.

Other common behavioral intervention strategies include reminder messages, personalization, and self-monitoring. Text message reminders are increasingly being used in health promotion interventions as a simple and cost-effective way to enhance healthy behaviors [83]. Several meta-analyses have demonstrated the efficacy of text message reminders across a wide range of populations and behaviors, with personalized messages being the most efficacious compared with generic messages or no messages at all [83,84]. Our previous work also suggests that using anchoring plan text message reminders in addition to the anchoring plan can lead to small, but significant, increases in meditation practice over an 8-week period [31]. Although the evidence to support the long-term effectiveness of text message reminders has been inconsistent [85], using personalized anchoring plan text message reminders may be a feasible, acceptable, and effective way to increase anchoring plan adherence [31,84].

Different types of self-monitoring messages may also influence anchoring plan adherence [86-88]. Self-monitoring has been cited as an important facilitator of behavior change and habit formation [74], and many existing mHealth apps have incorporated self-monitoring features to improve and sustain user engagement [89-91]. In addition, existing interventions have successfully used prompts either alone or in combination with other strategies to support health behavior change [92,93]. According to Michie et al [94], there are 2 types of self-monitoring: self-monitoring of behavior and self-monitoring of behavior outcomes. Messages that focus on self-monitoring of a target behavior (eg, number of completed meditation sessions during the past week and changes in the number of completed meditation sessions over time) may increase self-efficacy for a behavior by helping individuals visualize their effort investment and compliance with their behavioral goals [74]. Self-monitoring of behavior also helps individuals build self-regulatory skills [95], which may be particularly useful during the “learning phase” of the habit formation process [46]. Alternatively, messages that focus on self-monitoring of the outcomes associated with the meditation practice may increase the salience of both the immediate and long-term benefits of meditation practice, which may not be as clear from meditation practice alone [96,97]. In fact, our previous work on the Calm app’s self-monitoring mood check-in feature (ie, assessing and documenting mood before or after meditation practice) found that using mood check-ins was associated with increased meditation engagement, particularly among inactive subscribers [96]. As the mental health benefits of meditation are not immediately observable, messages that focus on the self-monitoring of outcomes of behaviors (ie, mood check-ins) may be a more effective way to reinforce participants’ adherence to the ≥10 minute-per-day meditation prescription than messages that focus on self-monitoring of behavior, which can only provide an indirect signal of participants’ mental health benefits. By varying the type of self-monitoring messages (ie, behavior or outcomes of behavior) between intervention study groups, we will be able to test the following hypothesis:

- *Hypothesis 1*: the study groups receiving messages that focus on self-monitoring of outcomes of meditation behavior will have greater adherence to the ≥10 minute-per-day meditation prescription during the 8-week intervention and during the 8-week postintervention observation periods than the study groups receiving messages that focus on self-monitoring of meditation behavior.

In addition to self-monitoring messages, offering financial or in-kind incentives may be a successful strategy to further increase anchoring plan adherence [98]. Research from the behavioral economics literature suggests that traditional external rewards are often successful in *initiating* a wide range of health behaviors [99-101]. While the effects of these behavioral economics–based incentives often disappear once the incentives are withdrawn [21,23], it is possible that combining incentives with the anchoring strategy may leverage the short-term effectiveness of incentives to increase anchoring plan adherence and establish long-term habits [24]. One way in which this combined approach could be implemented is by using “time-contingent” incentives that are conditional on performing the target behavior at approximately the same time that the planned anchor (ie, existing routine) is expected to occur. Such time-contingent incentives (TCIs) may direct participants’ focus and attention to the use of their anchors, which may help to better maintain meditation after the incentives are withdrawn. To test this approach and compare these strategies with the previous behavioral economics literature, the intervention study groups will receive either TCIs or non-TCIs (NTCIs) during the 8-week intervention period. This variation in intervention approaches will allow us to evaluate the following hypothesis:

- *Hypothesis 2*: The study groups receiving TCIs will have greater adherence to their anchoring plans and to the ≥10 minute-per-day meditation prescription during the 8-week intervention and during the 8-week postintervention observation periods than the study groups receiving NTCIs.

Combining anchoring, a well-studied habit formation strategy, with additional intervention strategies (ie, self-monitoring messages and incentives) is a novel behavioral intervention that may overcome adherence barriers and develop strong meditation habits. Therefore, our study will test anchoring in combination with varying forms of these 2 additional strategies to determine the optimal combined approach for establishing and maintaining meditation with a mindfulness meditation app. Importantly, our findings will test the following hypothesis regarding the success of the anchoring plan for maintaining the behavior change:

- *Hypothesis 3*: The study groups that display the greatest anchoring plan adherence during the 8-week intervention will be the most successful in maintaining meditation adherence during the 8-week postintervention observation period.

## Methods

### Study Setting

This study will be conducted using a nationwide sample of new, self-initiated Calm subscribers who purchased 1-year-long membership. There is no specified location for this study, as all study procedures can be completed remotely through our web-based surveys and the meditation app, and participants will not be required to complete any in-person laboratory procedures.

### Study Design

This study will use a 5-arm, parallel, partially blinded (participants only) randomized controlled design. After completing a web-based eligibility survey, the participants will be randomized into 1 of the 5 study groups. Stratified randomization methods will be used to ensure that race and ethnicity (ie, Hispanic) are equally represented across study groups. The 5 study groups include a control group that will use the anchoring plan to form a meditation habit with Calm and then receive no additional intervention supports, while the 4 intervention groups will also use the anchoring plan in combination with 1 of the 2 different types of self-monitoring messages and 1 of the 2 different forms of incentives. Participants in the 4 intervention groups will also be eligible for an in-kind reward (eg, Calm t-shirt, Calm tote bag, or Calm water bottle; all gifts have a retail value of roughly US $25 but a wholesale cost of US $5) conditional on either completing ≥10 minutes of meditation at any time of day on at least 8 days over a 2-week period (NTCIs) or completing ≥10 minutes of meditation within a 2-hour window around the time their chosen anchor typically occurs during the day (ie, anchor time1 hour) on at least 8 days over a 2-week period (TCIs). All additional interventions to support adherence to the anchoring plan will be delivered for 8 weeks; thus, participants are eligible to win up to 4 in-kind incentives during the intervention period. Participants within the intervention groups will also be randomly assigned to receive either messages that focus on self-monitoring of behavior (eg, “You completed a meditation session using your Calm app on at least 8 days during the past 2 weeks”) or messages that focus on self-monitoring of outcomes of behavior (eg, “We encourage you to continue using the ‘How Are You Feeling?’ check-in feature after completing your meditation sessions”). The “How Are You Feeling?” check-in feature is an existing feature available to all users of the Calm app that allows users to record their current mood state (eg, happy, sad, or anxious). The fractional factorial study design is shown in Table 1.

**Figure 1:**
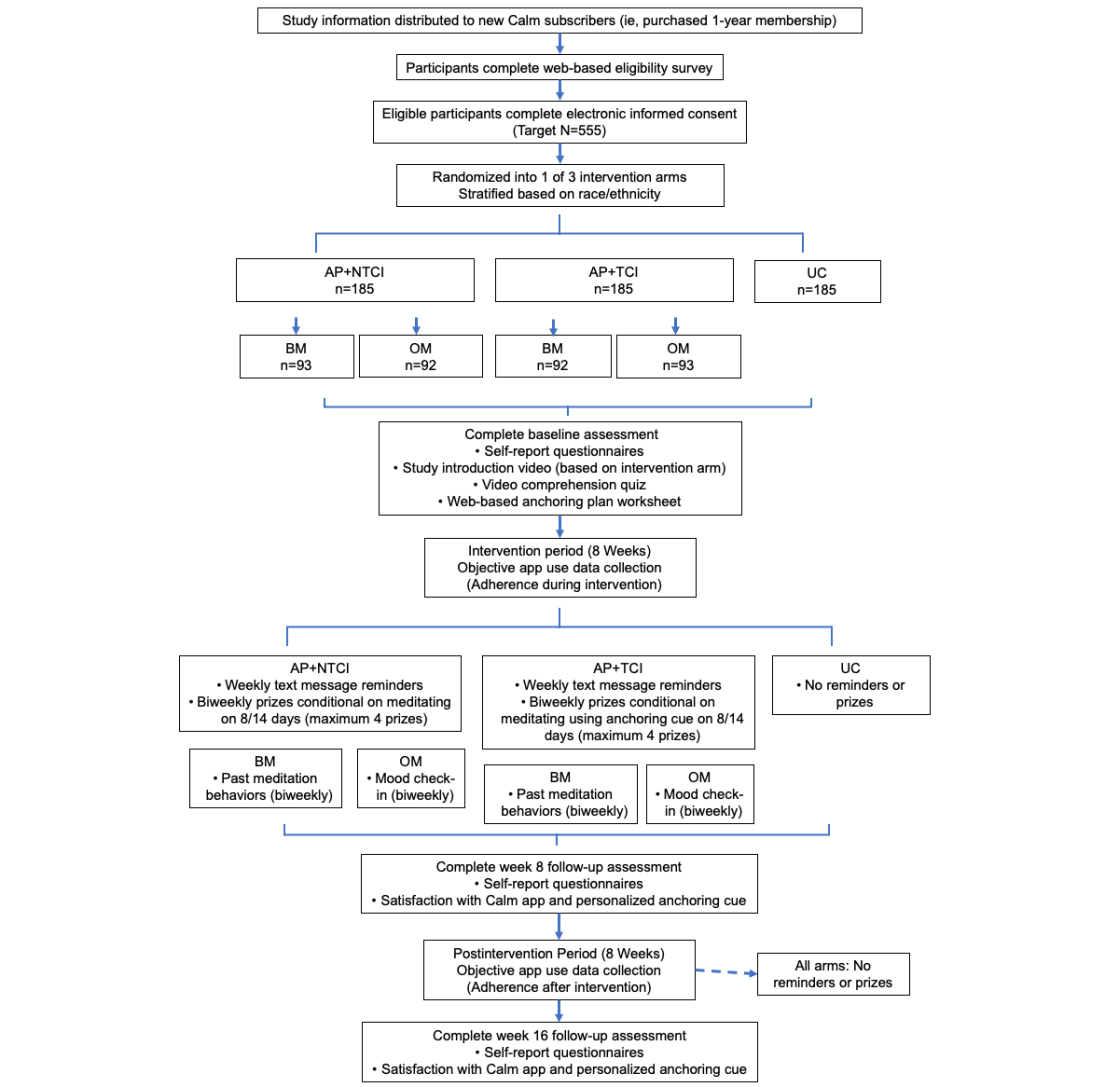
Timeline of study activities. AP: anchoring plan; BM: self-monitoring behavior; NTCI: non–time-contingent incentive; OM: self-monitoring outcomes; TCI: time-contingent incentive; UC: usual Calm.

After randomization, all participants will complete a baseline survey that includes self-report questionnaires, a study introduction video (described below under *Introductory Video*), a comprehension quiz, and a web-based worksheet that will guide the participants on how to develop their meditation anchoring plan. At weeks 8 and 16, participants will complete a follow-up survey to assess changes in our secondary outcome measures as well as satisfaction with the Calm app, their anchoring plan, and the intervention. Figure 1 outlines the timeline of all study activities.

**Table 1:** Fractional factorial study design^a^.

Study group	AP^b^	BM^c^	OM^d^	NTCI^e^	TCI^f^
Control	1	0	0	0	0
Treatment 1	1	1	0	1	0
Treatment 2	1	0	1	1	0
Treatment 3	1	1	0	0	1
Treatment 4	1	0	1	0	1

^a^This table shows which of the intervention strategies are received by each of the 5 study groups, where the column headers list each strategy and 1 indicates that the strategy is received by the study group listed in the row label.

^b^AP: anchoring plan.

^c^BM: self-monitoring of behavior messages.

^d^OM: self-monitoring of outcomes of behavior messages.

^e^NTCI: non–time-contingent incentive.

^f^TCI: time-contingent incentive (conditional on meditation within a 2-hour window around anchor timing).

### Sample Size

The target recruitment sample size for this study is 555 participants, with 111 participants per study group. On the basis of our existing studies using the Calm app, we anticipate a 20% study attrition, which will yield an effective study group sample size of 88. Our primary measure of meditation app adherence is a binary indicator variable for performing ≥10 minutes of meditation on at least 4 days of the week. This measure of adherence is based on the concept of behavioral regularity, which was proposed by Cheung et al [102] as a standardized measure of treatment adherence. In addition, researchers have recommended that practicing mindfulness for 3 to 4 days per week is the sufficient level of adherence (or regularity) for attaining the mental health benefits associated with meditation, such as stress reductions [103], and this recommendation is theoretically consistent with the notion that a mindful mind is the product of frequent, effortful, and persistent training [104,105]. Thus, we determined our sample size to detect study group differences in meditation regularity at the end of the study (week 16). Using data from our previous studies with Calm, we know that approximately 15% of new Calm users regularly meditate (ie, complete ≥10 minutes of meditation for at least 4 days of the week) 8 weeks after first subscribing to the app and fewer than 10% regularly meditate with Calm 16 weeks after subscribing. An effective study group sample size of 88 provides adequate statistical power (1–β=.82) for detecting study group differences of 18 percentage points in the likelihood of regular mediation in week 16 (Cohen *h*=0.43, which is a small to medium effect size).

### Eligibility

Participants will be eligible and included in our sample if they are aged at least 18 years, are new Calm subscribers (ie, have purchased at least 1-year of membership within the past week), are able to read and understand English, have access to a smartphone daily, reside in the United States or a US territory, and have a Perceived Stress Scale (PSS; PSS-4) score ≥6. Participants will be excluded if they are new Calm subscribers but have used Calm for ≥2 days in the last week, have practiced meditation (including meditative movement, ie, yoga or tai chi) for >60 minutes a month in the past 6 months, or are unable to provide consent or understand study procedures owing to mental illness or cognitive limitations.

### Recruitment and Enrollment Procedures

Potential participants will be invited to participate in the study via weekly recruitment emails sent by Calm to all new subscribers. The recruitment email will contain a link to complete an eligibility survey using the survey software Qualtrics (Qualtrics International Inc). Recruitment emails will be sent every week for 6 consecutive weeks or until the target sample size has been met. Participation will be voluntary, and participants may withdraw from the study at any time.

The eligibility survey will contain a brief overview of the study along with a series of questions based on the eligibility criteria listed in the previous section. If eligible, the participants will be asked to read an electronic consent form and provide their consent via electronic signatures. The electronic consent form also contains 2 comprehension questions to ensure that participants understand the study procedures. Once consented and enrolled, participants will be told that they will receive additional study instructions (ie, the baseline survey) via email in the coming weeks. During this time, eligible participants will be randomized based on the stratification dimensions described in the *Study Design* section.

### Data Collection Tools and Procedures

#### Overview

Data will be collected at 3 time points: baseline, week 8 (following completion of the intervention), and week 16 (following the 8-week postintervention observation period). Objective app use data will be collected via the Calm app, which will record the day and time of every app session; the duration of sessions; the type of session (eg, meditation, sleep story, and breathing exercise); and title of sessions (eg, Daily Calm and Daily Move) performed during the study period. Table 2 shows the primary and secondary outcome measures collected at each administration time point.

**Table 2:** Primary and secondary outcome measures at each administration time point.

Measure			Assessment		Baseline		8-week follow-up		16-week follow-up		Continuous data collection
**Primary outcomes**											
	Meditation adherence	Objective app use data		N/A^a^		N/A		N/A		Yes	
	Anchoring plan adherence	Objective app use data		N/A		N/A		N/A		Yes	
**Secondary outcomes**											
	Stress	Perceived Stress Scale		Yes		Yes		Yes		N/A	
	Anxiety	Hospital Anxiety and Depression Scale		Yes		Yes		Yes		N/A	
	PTSD^b^	Impact of Events Scale—Revised		Yes		Yes		Yes		N/A	
	Sleep disturbances	Insomnia Severity Index		Yes		Yes		Yes		N/A	
	Habit strength	Self-report Behavioral Automaticity Index		Yes		Yes		Yes		N/A	

^a^N/A: not applicable.

^b^PTSD: posttraumatic stress disorder.

#### Primary Outcomes: Adherence Maintenance (Objective App Use Data)

The primary outcomes for this study measure participants’ ability to maintain adherence to the ≥10 minute-per-day meditation prescription over the 16-week study period. Specifically, we will measure meditation adherence as a binary variable equal to 1 if participants complete ≥10 minutes of meditation for ≥4 days per week and 0 otherwise. This meditation adherence outcome will be defined weekly during both the 8-week intervention and the 8-week postintervention observation periods. We will also measure objective meditation app use using the number of days with ≥10 minutes of meditation per week and the total number of meditation minutes per week, both of which will allow us to investigate the behavioral frequency of app-based meditation and improve the statistical power of our analyses. We will also augment these objective app-based meditation measures with a self-reported measure of days with any meditation (ie, either using the app or meditation outside the app) over the past week that is assessed at baseline, week 8, and week 16 time points. We will measure anchoring plan adherence as a binary variable equal to 1 if participants complete ≥10 minutes of meditation within 1 hour of the timing of their chosen anchor for ≥4 days per week and 0 otherwise. Similar to overall meditation, we will also measure anchoring plan adherence as the number of days completing ≥10 minutes of meditation within 1 hour of the timing of their chosen anchor per week.

#### Secondary Outcomes: Stress, Anxiety, and Posttraumatic Stress Disorder

We will assess stress at baseline, week 8, and week 16 using the PSS-10. The PSS is a 10-item self-report inventory that assesses the degree to which individuals have felt stressed during the past month [106]. The PSS is a widely used measure of self-reported stress that has demonstrated acceptable validity and reliability in previous research [107]. We will also assess anxiety symptoms at baseline, week 8, and week 16 using the Hospital Anxiety and Depression Scale. The Hospital Anxiety and Depression Scale is a 14-item self-report inventory that assesses the severity of anxiety and depressive symptoms during the past week [108]. For our purposes, we will only be focusing on the anxiety subscale. Finally, we will assess posttraumatic stress disorder (PTSD) symptoms at baseline, week 8, and week 16 using the Impact of Events Scale—Revised. The Impact of Events Scale—Revised is a 22-item self-report inventory that assesses the severity of PTSD symptoms (eg, trouble sleeping, irritability, and jumpiness) over the past 7 days [109]. The instructions were modified to include PTSD symptoms resulting from specific trauma or from the ongoing COVID-19 pandemic.

#### Secondary Outcome: Sleep Disturbances

We will assess sleep disturbances at baseline, week 8, and week 16 using the Insomnia Severity Index (ISI). The ISI is a 7-item self-report inventory that assesses the severity of sleep problems, noticeability of sleep problems, stress resulting from sleep problems, and the perceived impact of these problems on daily functioning and quality of life [110]. The ISI is a valid and reliable measure that has demonstrated excellent internal consistency in previous research [111].

#### Secondary Outcome: Habit Strength

We will assess habit strength at baseline, week 8, and week 16 using the Self-Report Habit Index (SRHI). The SRHI is a brief self-report inventory that assesses the perceived habit strength (ie, automaticity) of a specific behavior, such as meditation [112]. The SRHI has been used to assess habit strength in a wide range of behaviors and has demonstrated adequate reliability in previous research [112].

#### Demographic Information

The following demographic information will also be collected from participants at baseline assessment: age; gender; race; Hispanic status; employment information (ie, status, hours, and work from home); annual household income; number of adults and children in the household (if any); marital status; education level; past mental health diagnosis (ie, PTSD and depression); current mental health medications (if any); self-rated physical health (5-point Likert scale); and any current chronic medical condition diagnosis (eg, high blood pressure, diabetes, and asthma).

#### Satisfaction

Participant satisfaction will be assessed at weeks 8 and 16. All participants will be asked to rate their overall enjoyment and satisfaction with the Calm app and the study itself, as well as their intent to continue using the app along with the app features (eg, mood tracking and session tracking) and app content (eg, daily meditations, sleep meditations, and soundscapes) that they found most helpful for maintaining their daily meditation practice. Then, participants will be asked specific questions regarding their chosen meditation anchor, such as how easy it was to select and use their chosen anchor during the intervention, their intent to continue using their meditation anchor, and the helpfulness of the anchoring plan in maintaining their meditation practice. Participants in the intervention groups will also be asked questions related to the incentives, including how easy it was to understand the incentive process; how easy it was to earn incentives; if they liked the incentive options, how motivated they were by the incentives; and if there was anything that would have motivated them more. This information will provide helpful qualitative data that can be used to improve future adherence studies, including the types of anchoring strategies that are most successful in establishing a meditation habit and what motivates people to establish and maintain an app-based meditation habit.

Attrition will be assessed at all stages of the study: (1) enrolled but did not complete the baseline assessment, (2) completed baseline assessment but lost to follow-up during the 8-week intervention period (ie, did not complete week 8 assessment), and (3) completed the 8-week assessment but lost to follow-up during the 8-week postintervention observation period (ie, did not complete the week 16 assessment).

### Intervention

#### Randomization and Baseline

This randomized controlled trial aims to test the effectiveness of different strategies on adherence to the daily ≥10-minute meditation prescription via a mindfulness meditation app. Note that the meditation prescription and the anchoring plan are not interventions; rather, the interventions are the additional supports that are provided for the anchoring plan to establish an app-based meditation habit. After randomization, all participants will complete a web-based baseline survey to collect additional demographic information and to assess the baseline levels of the secondary outcome measures listed in the previous sections.

#### Introductory Video

Immediately following the web-based surveys, participants will then watch 1 of 3 brief 5-minute introductory videos that will vary slightly based on their experimental conditions. All 3 videos will contain background information on the benefits of mindfulness meditation and habit formation (eg, previous research and theory); a sample anchoring plan (eg, “After [insert behavior here], I will meditate for 10 minutes”); a study timeline (ie, baseline assessment, 8-week intervention period, follow-up assessment, 8-week postintervention period, and second follow-up assessment); and compensation information for completing the 3 web-based surveys (US $20 gift card). In addition to these foundation elements, participants in the NTCI and TCI groups will be told in the video that they are eligible for incentives every 2 weeks during the 8-week intervention period conditional on either meditating at any time (NTCIs) or meditating using their anchoring plan (TCIs). The video for the usual Calm condition will only include the foundation elements mentioned previously, with no mention of incentives or personalized feedback messages. Information in all 3 videos will be presented by a member of the study team using both written and spoken texts. Participants in the NTCI and TCI groups will also receive instructions regarding prize eligibility as detailed in the *Study Design* section.

#### Anchoring Plan Worksheet

After watching the introductory video, participants will complete a video comprehension quiz to ensure that they understand the study timeline and requirements. Participants must score 100% on the quiz to move on with the study. Then, all participants will be asked to complete a web-based worksheet to help them develop a meditation anchoring plan. This worksheet will first ask participants to fill out a basic implementation intention plan (ie, “After _____, I will meditate for 10 minutes”) based around a routine behavior (ie, their anchor) they perform in the morning, given that previous research suggests the morning habits are easiest to form [31]. Next, participants will be instructed to add additional details to their anchoring plan to make it more specific (eg, anchor timing and anchor location), as generic plans may not be as effective as plans that specifically outline when and where this behavior will occur. After finalizing their anchoring plan, the participants will be emailed a copy of their anchoring plan for their records.

#### Intervention, Postintervention, and Follow-up Surveys

The 8-week intervention period will begin once the participants complete the baseline assessment and anchoring plan worksheet. All the participants will be asked to meditate for ≥10 minutes every day during the intervention period. Every 2 weeks, participant use data will be checked to determine incentive eligibility for participants in the NTCI and TCI groups. Every week, all the participants will also receive a text message from the study team as a reminder to continue meditating using their chosen anchor. In addition, participants in the BM group will receive a message focused on self-monitoring of their meditation behavior every 2 weeks that informs participants of the number of ≥10-minute meditations they completed during the past 2 weeks. Participants in the self-monitoring of outcomes of behavior messages groups will receive a message focused on self-monitoring of outcomes of their meditation behavior (ie, mood tracking) every 2 weeks that encourages the participants to keep track of their mood using the “How Are You Feeling?” feature in the Calm app. After 8 weeks, participants will be emailed a link to complete the first of the 2 follow-up surveys. These surveys will assess any improvements in the secondary outcome measures assessed at baseline, as well as their satisfaction with both the Calm app itself and the interventions. Following the completion of the 8-week intervention period and the week-8 follow-up survey, participants will be asked to continue using their anchors to strengthen their meditation habits during the 8-week postintervention observation period. However, unlike in the intervention period, participants in all intervention groups will no longer receive weekly reminders, biweekly self-monitoring messages, or incentives during the postintervention period. Participants will then be contacted at week 16 to complete the second follow-up survey to assess changes in the secondary outcome measures at week 16.

### Data Analysis Plan

To examine our specific aims, statistical analyses comparing group-level differences in the primary and secondary outcome measures will be performed. First, an analysis of covariance framework will be used to test for group-level differences in each primary and secondary outcome, controlling for participant characteristics that are found to significantly differ between study groups at baseline. For analyses of dichotomous outcomes, such as meditation regularity at weeks 8 and 16, a nonparametric McNemar test and multiple logistic regression that controls for additional participant-level covariates will be used to assess study group differences. In addition to static comparisons of group means for each outcome, the longitudinal nature of the app use data will be leveraged by using repeated measures and time-series techniques. Specifically, a linear mixed model with repeated observations will be fitted using maximum likelihood through “xtmixed” in the software package Stata (StataCorp) to study group-level temporal dynamics in the primary and secondary outcomes.

To estimate the impact of our intervention on our static primary outcomes of meditation regularity and anchoring plan regularity at weeks 8 and 16, we will use logistic regressions with the following specifications.

Our main unadjusted model will have the following form:

Pr(y_i_) = exp(*β*_0_+ *β*_1_T + *β*_2_T2 + *β*_3_T3 + *β*_4_T4 + ε_i_  **(1)**

Our main adjusted model has the following form:

Pr(y_i_) = exp(*β*_0_+ *β*_1_T + *β*_2_T2 + *β*_3_T3 + *β*_4_T4 + X_i_
*β*_5_ + ε_i_  **(2)**

where *y_i_* is a binary variable equal to 1 if individual i completed ≥10 minutes of meditation for ≥4 days during week 8 of the study; T1 is an indicator for treatment group 1 (that receives self-monitoring behavior messages and NTCIs); T2 is an indicator for treatment group 2 (that receives self-monitoring outcome messages and NTCIs); T3 is an indicator for treatment group 3 (that receives self-monitoring behavior messages and TCIs); T4 is an indicator for treatment group 4 (that receives self-monitoring outcome messages and TCIs); and *ϵ_i_* is the idiosyncratic error. The coefficients of interest are *β*_1_ through *β*_2_. Equation 2 has the term *X_i_β*_5_, which includes a vector of the following observable participant characteristics (*X_i_*): age, education, sex, race, ethnicity, household income, self-reported mental and physical health, baseline meditation habit strength, intrinsic motivation for meditation, and measures of time and risk preferences and the respective coefficients (*β*_5_). For more information on our data analysis plan, please see Multimedia Appendix 1.

In addition, we will investigate potential intervention moderators and mediators. Specifically, we will use the same regression models specified earlier in this section and include additional interaction terms between each study group indicator variable and a single moderating variable measured at baseline. Significant coefficients estimated for these interaction terms will be used to identify the moderating effects of participants’ baseline characteristics, such as age, gender, race, and employment information. In addition, we will separately examine the mediating effects of anchoring plan adherence and habit strength at the end of the intervention on the maintenance of mediation following the procedures outlined by Judd and Kenny [113]. We will first regress meditation measured in week 16 on indicator variables for each study group and then regress meditation in week 16 on indicator variables for each study group as well as anchoring plan adherence measured over the first 8 weeks (ie, during the intervention). The mediating effect of anchoring plan adherence (our third hypothesis) will be calculated by subtracting the coefficients estimated in the first regression from those in the second regression. We will also use these procedures to investigate the mediating effect of habit strength measured at week 8 (ie, immediately after the intervention). We will also use a modern mediation analysis technique that allows for additional covariates when estimating the direct and indirect treatment effects in the equations described earlier [114].

### Ethics Approval

This research was reviewed and approved by the Arizona State University Institutional Review Board (STUDY00014710) on 11/9/2021. This study has also been registered at ClinicalTrials.gov NCT05217602. Participants will provide consent electronically and will be emailed a copy of the consent form for their records. Participants can withdraw from the study at any time without penalty and request that their data be removed from the analysis. The data collected by the research team will be stored on a password-protected cloud-based secure server. All data retrieved from the questionnaires will be deidentified (kept separate from any personal identifying information, including consent forms). The questionnaire data will only be linked to individual participants via the assigned study ID numbers. Only anonymous aggregated data regarding app use during the 8-week study and an additional 8-week postintervention observation period will be retrieved from the Calm informatics team. All the participants will be compensated US $20 in electronic gift cards for completing the 3 web-based surveys (ie, baseline, postintervention, and follow-up surveys). Participants in either the NTCI or TCI groups will also be eligible to win up to 4 incentives during the intervention period, in addition to a US $20 gift card as compensation for completing all 3 web-based surveys.

## Results

Recruitment for this study was expected to occur between March 2022 and May 2022. Data collection is expected to be completed by October 2022. The results from this study will be available within 12 months of the final data collection date, which will be sometime between late 2022 and early 2023. The findings of this research will be disseminated using various methods, including peer-reviewed journals, academic conferences, and other verbal and digital communication platforms.

## Discussion

### Overview

Mobile meditation apps can be an effective and feasible way to deliver mindfulness meditation interventions. However, low intervention adherence over time limits the long-term potential of these apps. This study aims to address this critical issue by testing a combination of behavioral intervention strategies to determine the optimal combined strategy for establishing and maintaining meditation habits with a mobile app. To accomplish this, we have three specific aims: (1) assess study group differences in adherence to the daily meditation prescription during the 8-week intervention, (2) estimate study group differences in adherence maintenance over the 8-week postintervention observation period, and (3) investigate the role of anchoring performance during the intervention on the participants’ ability to maintain their meditation app adherence during the postintervention observation period.

This study will make important contributions to mHealth and behavior change research. First, the results of this study will improve future app-based interventions by identifying the most effective strategies for establishing and maintaining adherence to a mobile app, adding to the sparse body of literature on adherence to app-based interventions, particularly those for mindfulness meditation. Current research on app-based interventions is limited by poor adherence, and improving adherence is necessary to understand the full potential of app-based interventions for promoting health behavior change as well as the associated benefits from these health apps. This is particularly important for health behaviors such as mindfulness meditation, which requires maintaining a long-term practice to experience the maximum physical and mental health benefits. Second, this study will assess adherence by using objective app use data. Previous studies examining adherence in app-based interventions have found significant discrepancies between self-reported (eg, retrospective, daily diaries) and objective measures of adherence, as self-report measures are subject to more biases such as recall or response bias [115]. Testing strategies for maintaining objective measures of app-based intervention adherence will provide more rigorous evidence that will help to better understand the habit formation process in this setting. Finally, this research will make important contributions to the behavior change literature, in which existing interventions in the psychology and behavioral economics literature have largely been unsuccessful in maintaining behavior changes after interventions. By combining the anchoring habit formation strategy from psychology with behavioral economics–based incentives (ie, in-kind prizes awarded for short-term behaviors), this study will provide important evidence for a novel combined approach that may be more successful in maintaining behavior changes and will help improve our understanding of the habit formation process.

Several potential limitations of this study must also be addressed. First, the 8-week intervention period may not be sufficient to develop a strong and long-lasting meditation habit. Similarly, the 8-week intervention and 8-week postintervention periods may not be long enough to adequately assess our primary and secondary outcomes. Future research should plan to assess adherence and our secondary outcomes over longer periods to better understand the long-term effectiveness of these strategies. Another limitation to consider is the likely demographic characteristics of the recruited sample. Previous studies using Calm have reported samples that consisted of predominantly individuals who are White, female, and have bachelor’s degrees or higher qualification [116,117]. As a result, we anticipate having difficulty recruiting a diverse sample, which will limit the generalizability of our findings. In addition, studying the meditation app behavior of paid subscribers to a meditation app may not be generalizable to the general population. We plan to address this limitation by using a stratified randomization method to ensure that each study group has equal representation across racial and ethnic groups. Finally, it is possible that the incentives (ie, Calm prizes) provided to the NTCI and TCI groups during the intervention period may not be incentivizing to participants. We will test this by comparing the NTCI and TCI groups with the control group and by examining which items were most commonly chosen by eligible participants, and we will publish the results of these analyses to help determine the magnitude of incentives in future incentive-based meditation studies.

### Conclusions

This study will contribute to an important gap in the literature by testing novel intervention approaches for establishing and maintaining adherence to app-based mindfulness meditation. We are using unique combinations of previously established intervention strategies from the psychology and behavioral economics literature to help participants better maintain adherence to an effective dose of meditation that can reduce stress. If successful, this study will identify an accessible and scalable stress self-management intervention to combat the ongoing stress epidemic in the United States.

## Appendix

Multimedia Appendix 1 Data analysis plan.

## Abbreviations

ISI: Insomnia Severity Index
mHealth: mobile health
NTCI: non–time-contingent incentive
PSS: Perceived Stress Scale
PTSD: posttraumatic stress disorder
SRHI: Self-report Habit Index
TCI: time-contingent incentive
