# Supplementary material for: Combining Behavioral Economics–Based Incentives With the Anchoring Strategy: Protocol for a Randomized Controlled Trial
Source: JMIR Res Protoc. 2023 Apr 28;12:e39930. doi: 10.2196/39930 (PMC10182474; doi:10.2196/39930)
Supplement: Multimedia Appendix 1 [file resprot_v12i1e39930_app1.docx]

**Multimedia Appendix 1**

Additional Data Analysis

We will examine the impact of our intervention on weekly measures of our primary outcomes of meditation and anchoring plan adherence over the 16-week study period. We will analyze the temporal changes in these weekly outcomes using the following logistic regression model specification:

${Pr(y}_{it})=\exp(\beta_{0}+\sum_{t=1}^{16} \delta_{t}\left( {T1}_{it}\times W_{t} \right)+\sum_{t=1}^{16} \gamma_{t}\left( {T2}_{it}\times W_{t} \right)++\sum_{t=1}^{16} \alpha_{t}\left( {T3}_{it}\times W_{t} \right)+\sum_{t=1}^{16} \eta_{t}\left( {T4}_{it}\times W_{t} \right)+\sum_{t=1}^{16} W_{t}+\epsilon_{it} )$ (3)

where $y_{it}$ is either the weekly measure of meditation adherence or anchoring plan adherence in week t for individual i, $\delta_{t}$ captures the impact of T1 in week t, $\gamma_{t}$ captures the impact of T2 in week t, $\alpha_{t}$ captures the impact of T3 in week t, $\eta_{t}$captures the impact of T4 in week t, and $\epsilon_{it}$ is the idiosyncratic error. A similar regression framework will be estimated using a negative binomial model for assessing study group differences in the primary outcome of the number of days with ≥10 minutes of meditation, and an ordinary least squares model will be used to estimate treatment effects on the total number of meditation minutes per week.

We will use a similar set of static and dynamic regression models to assess changes in our secondary outcomes during the study. Additionally, to evaluate the impact of anchoring plan adherence on participants’ ability to maintain meditation adherence during the eight-week post-intervention period, the following logistic regression model will be used:

Our main unadjusted model will have the following form:

${Pr(y}_{i})=\exp(\beta_{0}+\beta_{1}T1+\beta_{2}T2+\beta_{3}T3+\beta_{4}T4+AP+\beta_{5}(T1\cdot AP)+\beta_{6}(T2\cdot AP)+\beta_{7}(T3\cdot AP)+\beta_{8}(T4\cdot AP)+ \epsilon_{i})$ (4)

and our main adjusted model has the following form:

${Pr(y}_{i})=\exp(\beta_{0}+\beta_{1}T1+\beta_{2}T2+ {\beta_{3}T3+\beta_{4}T4+AP+\beta_{5}\left( T1\cdot AP \right)+\beta_{6}\left( T2\cdot AP \right)+\beta_{7}\left( T3\cdot AP \right)+\beta_{8}\left( T4\cdot AP \right)+ \boldsymbol{X}}_{\boldsymbol{i}}\boldsymbol{\beta}_{9}+\epsilon_{i})$ (5)

where $y_{i}$ is a binary indicator of meditation adherence at week 16, TX is an indicator for treatment group X, AP is an indicator variable equal to one if anchoring plan adherence was above the median level of adherence during week eight, and TX • AP is an interaction term between the indicator for treatment group X and AP. The coefficients of interest are $\beta_{5}$ - $\beta_{8}$. Equation 5 includes the same set of participant characteristics as described in equation 2 above. We will similarly use a negative binomial model to assess study group differences in the number of days with anchored meditation.
